# Monitoring phosphorylation and acetylation of CRISPR-mediated HiBiT-tagged endogenous proteins

**DOI:** 10.1038/s41598-024-51887-x

**Published:** 2024-01-25

**Authors:** Juliano Alves, Marie Schwinn, Thomas Machleidt, Said A. Goueli, James J. Cali, Hicham Zegzouti

**Affiliations:** R&D Department, Promega Corporation, 2800 Woods Hollow Road, Madison, WI 53711 USA

**Keywords:** Biological techniques, Biotechnology, Cell biology, Drug discovery

## Abstract

Intracellular pathways transduce signals through changes in post-translational modifications (PTMs) of effector proteins. Among the approaches used to monitor PTM changes are immunoassays and overexpression of recombinant reporter genes. Genome editing by CRISPR/Cas9 provides a new means to monitor PTM changes by inserting reporters onto target endogenous genes while preserving native biology. Ideally, the reporter should be small in order not to interfere with the processes mediated by the target while sensitive enough to detect tightly expressed proteins. HiBiT is a 1.3 kDa reporter peptide capable of generating bioluminescence through complementation with LgBiT, an 18 kDa subunit derived from NanoLuc. Using HiBiT CRISPR/Cas9-modified cell lines in combination with fluorescent antibodies, we developed a HiBiT-BRET immunoassay (a.k.a. Immuno-BRET). This is a homogeneous immunoassay capable of monitoring post-translational modifications on diverse protein targets. Its usefulness was demonstrated for the detection of phosphorylation of multiple signaling pathway targets (EGFR, STAT3, MAPK8 and c-MET), as well as chromatin containing histone H3 acetylation on lysine 9 and 27. These results demonstrate the ability to efficiently monitor endogenous biological processes modulated by post-translational modifications using a small bioluminescent peptide tag and fluorescent antibodies, providing sensitive quantitation of the response dynamics to multiple stimuli.

## Introduction

Intracellular signaling pathways regulate different cellular processes fundamental to cell physiology through post-translational modifications. Disruption of these regulatory mechanisms can lead to pathological processes resulting in diseases such as cancer and inflammatory diseases^1,2^. A better understanding of these cellular processes would help elucidate the underlying mechanisms of disease as well as enable the discovery of novel pharmacological targets. Antibody-based assays and mass spectrometry are commonly used methods for quantifying post-translational modifications of endogenous proteins in complex biological systems. Although ELISA and Western Blotting are relatively easy and affordable methods, they are limited by the dynamic range and low throughput, as well as the availability of high-quality antibodies^3^.

Similarly, mass spectrometry-based methods are limited by the low throughput and the requirement of specialized instrumentation and techniques^4^. Alternatively, protein overexpression using expression vectors regulated by strong transcriptional promoters are also used to gain insight into the target function within a cellular environment. However, bypassing the endogenous genetic regulatory elements for protein expression could lead to artifacts as a result of unregulated protein expression^5^. Genome editing through CRISPR/Cas9 has become an important tool that facilitates the site-specific modification of target genes in a simple and easy-to-use format^6^.

The complementing subunits of a two subunit NanoLuc reporter system for measuring intracellular protein–protein interactions was reported, in which 11-amino acid peptide (SmBiT) interacts with an 18 kDa polypeptide (LgBiT) with varying affinities to generate an active luciferase that generates light with its luminogenic substrate furimazine^7^. One peptide in particular (HiBiT) has high affinity to LgBiT (K_D_ = 700 pM) and was evaluated as a reporter for tagging endogenous HIF-1α using CRISPR/Cas9 RNP complexes together with a ssODN homology-directed repair template^8^.

The high integration efficiency and assay sensitivity of HiBiT enabled quantitation of protein levels within 24–48 h after cell editing in mixed population of edited cells. This methodology enabled the monitoring of dynamic changes of endogenous HIF-1α (HIF-1α-HiBiT) levels in response to hypoxic conditions as well as to compounds that affect HIF-1α stability^8^. In addition, HIF-1α hydroxylation was efficiently monitored through bioluminescence resonance energy transfer (BRET) between the bioluminescence generated by HiBiT/LgBiT reconstituted luciferase and a fluorescently labeled antibody recognizing HIF-1α hydroxylation. Because BRET corresponds to a ratio of signals from the energy donor (i.e. luciferase) and the fluorescent acceptor, it indicates the degree of modification normalized to protein abundance in a specific manner. It should be noted that non-specific background signals are minimized in this system because antibodies bound to off-target proteins are nonproductive for energy transfer due to the absence of luciferase as the donor partner^8^. Moreover, the BRET assay was validated in comparison to standard methods, showing that the BRET results mirror exactly western blotting data related to the detection of HIF1α Pro564 hydroxylation and total HIF1α protein abundance^8^. While Ab-based detection by direct fluorescence is useful for western blotting, ELISA, and immunohistochemistry with multiple background blocking and washing steps, the BRET technology described here enables a sensitive, homogeneous, “add and read” only approach.

Building on the previous efforts toward detecting HIF-1α hydroxylation as well as hydroxylation-mediated degradation through BRET, here we demonstrate the feasibility of using a HiBiT-BRET immunoassay approach (i.e.: Immuno-BRET) to monitor several post-translational modifications and their regulation in selected disease-related pathways such as EGFR, JAK/STAT, MAPK8 and c-MET (phosphorylation), as well as dynamic changes in histone H3 acetylation. Since the coupling of genome editing through CRISPR/Cas9, fluorescently labeled antibodies and HiBiT/LgBiT reconstitution enabled the monitoring of intracellular signaling pathways through detection of post-translational modifications, our results suggest that these tools could have significant impact in areas such as cell signal transduction and epigenetics research.

## Materials and methods

### Cells and reagents

Human recombinant EGF, digitonin (100 ×), Nano-Glo HiBiT Lytic Buffer, Nano-Glo HiBiT Lytic Substrate, and LgBiT Protein were from Promega Corporation. The compounds trichostatin-A, ruxolitinib, anisomycin, SP 600125 and crizotinib were from TOCRIS. The secreted factors IL-4, CNTF, IL-27, CLC, IL-6, LIF, CT1, IFN-γ, M-CSF, TNF-α, BMP-6, SDF-1, IL-1-β/IL-1 F2, IL-1-α/IL-1F1, MD2, IL-8/CXCL8, HGF, TGF-β, Inhibin, Activin, Nodal, GDF-1 and GM-CSF were purchased from R&D Systems. The antibodies anti p-EGFR (Y1045), anti p-EGFR (Y1068), anti p-EGFR (Y1173), antiphospho-tyrosine (P-Tyr-1000) MultiMab, anti p-STAT3 (Y705), anti p-SAPK/JNK (T183/Y185), anti p-MET (Y1349), anti p-MET (Y1234/1235), anti ac-histone H3 (K9), anti ac-histone H3 (K27), and anti-rabbit IgG (H + L), F(ab')2 Fragment (Alexa Fluor^®^ 594 Conjugate) were obtained from Cell Signaling Technologies. 100 × HALT Protease/Phosphatase Inhibitor Cocktail was purchased from Thermo Fisher. Dulbecco’s Modified Eagle Medium (DMEM), IMDM, Pen/Strep were from Gibco. Fetal bovine serum (FBS) was purchased from VWR. HeLa (CCL-2) cells were purchased from American Type Culture Collection. Alt-R S.p. Cas9 Nuclease V3, Alt-R CRISPR-Cas9 tracrRNA, Alt-R CRISPR-Cas9 crRNA, Ultramer DNA Oligonucleotides (ssODN donor DNA templates), and Nuclease-Free Duplex Buffer were from Integrated DNA Technologies. HeLa and A-432 cells were grown in DMEM/10% fetal bovine serum/1% Pen/Strep at 37 °C with 5% CO_2_. K-562 cells were grown in IMDM/10% fetal bovine serum at 37 °C with 5% CO_2_. COSTAR white 96-well tissue culture plates were from Corning.

### CRISPR-mediated knock-in

Guide RNA (gRNA) were generated by combining 1.2 nmol Alt-R tracrRNA with 1.2 nmol Alt-R crRNA in a final volume of 50 µL Nuclease-Free Duplex Buffer and heating at 95 °C for 5 min. Ribonucleoprotein (RNP) complexes were generated by incubating 120 pmol gRNA and 100 pmol Cas9 for 10 min at ambient temperature. 2 × 10^6^ cells were resuspended in 100 µL of Ingenio Electroporation Solution (Mirus) and RNP complex plus 100 pmol donor DNA template were added to cell solution. Cells were electroporated at 130 V and returned to complete growth medium for an additional 24–48 h before assaying for bioluminescence. To isolate clones, pools of edited cells were resuspended to 5 × 10^6^ cells in 1 mL sorting buffer (Hank’s Balanced Salt Solution, 10 mM HEPES, 0.2% bovine serum albumin, and 10 units penicillin–streptomycin), passed through a 35 µm mesh filter, and dispensed as single cells into a 96 well plate containing 150 µL of growth medium using the FACSMelody (BD Biosciences). When colonies were established (approximately 3 weeks), positive clones were identified using bioluminescence.

The sequences of the gRNA used to generate the knockin cell lines is as follows: H3F3A: GTAAGGAGGTCTCTGTACCA, MAPK8: TTGACAGACGACGATGATGA, MET: CACACGACCAGCCTCCTTCT, EGFR: AATTTATTGGAGCATGACCA, and STAT3: CCCATGTGAGGAGCTGAGAA. The sequences of the donor DNA templates are as listed with HiBiT integration sequence underlined: H3F3A: TGATTTTTGATTTTTCAATGCTGGTAGGTAAGTAAGGAGGTCTCTGTACCATGGTCTCCGTGAGCGGCTGGCGGCTGTTCAAGAAGATTAGCGCTCGTACAAAGCAGACTGCCCGCAAATCGACCGGTGGTAAAGCAC, MAPK8: CTGTCTGCAACTGATTTGCTGTTTTGTTTCTCATAGCACAGGTGCAGCAGGTCTCCGTGAGCGGCTGGCGGCTGTTCAAGAAGATTAGCTGATCAATGGCTCTCAGCATTCATCATCATCGTCGTCTGTCAATGATGTGTCT, MET: ACGCTGATGATGAGGTGGACACACGACCAGCCTCATTCTGGGAGACATCAGTCTCCGTGAGCGGCTGGCGGCTGTTCAAGAAGATTAGCTAGTGCTAGTACTATGTCAAAGCAACAGTCCACACTTTGTCCAATGGTTTTTT, EGFR: ATGCAGAATACCTAAGGGTCGCGCCACAAAGCAGTGAATTTATTGGAGCAGTCTCCGTGAGCGGCTGGCGGCTGTTCAAGAAGATTAGCTGACCACGGAGGATAGTATGAGCCCTAAAAATCCAGACTCTTTCGATACCCAG, and STAT3: CCCTCACCTTTGACATGGAGTTGACCTCGGAGTGCGCTACCTCCCCCATGGTCTCCGTGAGCGGCTGGCGGCTGTTCAAGAAGATTAGCTGAGGAGCTGAGAACGGAAGCTGCAGAAAGATACGACTGAGGCGCCTACCTGC.

### Detection of HiBiT tag

HiBiT fusion proteins were detected using the Nano-Glo HiBiT Lytic Detection System (Promega N3030) according to manufacturer’s protocol. Briefly, cells were plated in 96-well solid white assay plates (Corning 3917) at a density of 20,000 cells/well in a total volume of 100 µL culture medium. An equivalent volume of Nano-Glo HiBiT Lytic Reagent was added and the plates were placed on an orbital shaker for 3 min (300 rpm). Bioluminescence was measured on a GloMax Discover (Promega) with 0.3 s integration time.

### Immuno-BRET assay for analysis of post-translational modifications

CRISPR/Cas9-modified cells resuspended in culture media containing FBS were plated in 50 μL volumes in white 96-well tissue culture plates and incubated for 24 h at 37 °C. For detection of histone H3 acetylation, the following day cells were treated with serial dilutions of the histone deacetylase inhibitor trichostatin A and incubated at 37 °C overnight. For detection of phosphorylation events, the next day starvation was induced by replacing the media with serum-free media, and the assay plates were then incubated at 37 °C overnight. The following day cells were treated with serial dilutions of pathway stimulating ligands or inhibitors, and then lysed by the addition of 50 µL assay buffer (25 mM NaCl, 150 mM Tris–HCl at pH 7.5, 5 mM EDTA and 1 × HALT Protease/Phosphatase Inhibitor Cocktail) supplemented with 1 × digitonin, and rabbit anti specific phospho sites or anti-phosphotyrosine antibodies in addition to 100 nM LgBiT. Cells were incubated for 60 min at room temperature, followed by the addition of 100 μL assay buffer containing Alexa Fluor 594 labeled anti-rabbit secondary antibody (1:500 dilution, Cell Signaling Technology) and 100 μM furimazine, followed by incubation for 60 min at room temperature. Bioluminescence and fluorescence were then measured using a plate reader.

### Signal detection and data analysis

All 96-well assay plates were read using a multimode GloMax^®^ Discover Microplate Reader from Promega. The instrument was set to record both total bioluminescence and BRET signals (acceptor channel 460/60 nm, donor channel 610 nm long pass) using 0.5 s integration time. BRET ratio values were obtained by dividing the acceptor emission value by the donor emission value for each sample. Data plotting and analysis was performed using both Microsoft Excel and GraphPad Prism^®^, version 9 Software. IC_50_ values were determined by using a nonlinear regression fit to a sigmoidal dose response (variable slope).

## Results and discussion

**Figure 1:**
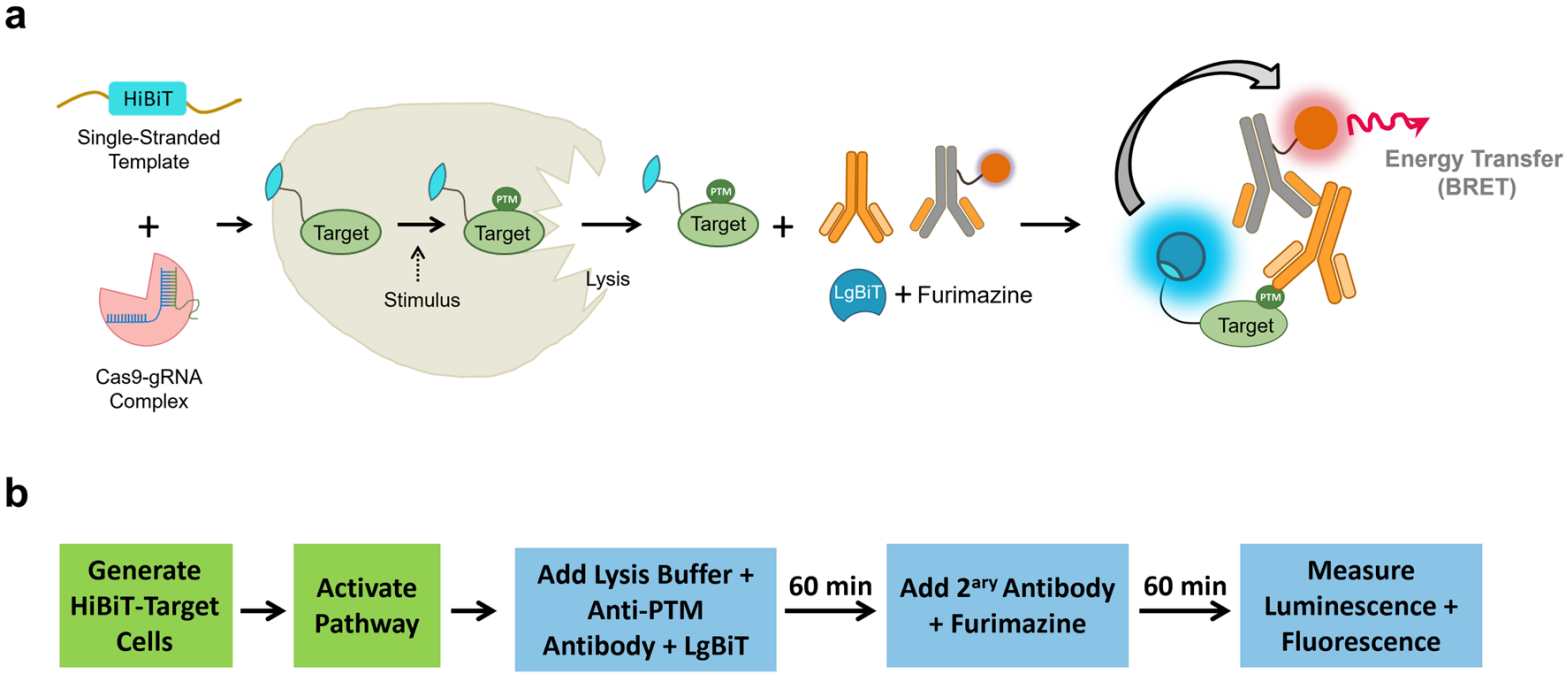
Immuno-BRET assay. (**a**) Principle of Immuno-BRET assay for PTM detection. After generating a HiBiT-target CRISPR/Cas9-modified cell line, cells are lysed and a PTM (e.g. phosphorylation) on the target protein is detected using a primary antibody specific for the PTM, a fluorophore conjugated secondary antibody and the LgBiT. The bioluminescence resonance energy transfer (BRET) is generated between the reconstituted NanoLuc (HiBiT-LgBiT) with its luminogenic substrate, furimazine, and the fluorophore only when they are in proximity due to the presence of the PTM. (**b**) Workflow of Immuno-BRET assay. A homogenous and simple “Add and Read” cell-based assay format.

CRISPR-mediated HiBiT tagging of endogenous proteins was shown to be broadly applicable to the study of numerous proteins in the human proteome^9^. Our work expands on previous findings describing a scalable method for HiBiT tagging of endogenous proteins to generate stable cell lines, consisting of integrating a HiBiT sequence upstream of the native stop codon for all targets. Whenever possible, cut sites within the 3’ untranslated regions were preferable to avoid potential NHEJ repair. Successful integration of the HiBiT tag was determined 72 h by measuring bioluminescence in cell lysates in the presence of LgBiT. As a result, off-target integration of HiBiT is extremely unlikely since it would require in-frame insertion into a protein encoding gene. To demonstrate the utility for monitoring dynamic changes in post-translational modifications in CRISPR/Cas9-HiBiT-tagged cells using Immuno-BRET assays, which combine BRET generated between fluorescently labelled antibodies and HiBiT/LgBiT complementation (Fig. 1), we focused on the cancer-related intracellular pathways regulated by the kinases EGFR, JAK/STAT, JNK and c-MET. The corresponding genes for these targets were edited using CRISPR/Cas9 for the insertion of the HiBiT peptide, to expand on the previously published study that used HiBiT tagging to monitor protein abundance to now monitoring endogenous protein modifications^9^. All the edited cell lines were stably expressing the HiBiT tag. The targets STAT3, MET and MAPK8 as well as their respective guide RNA and donor DNA sequences have been previously validated^9^. In this study we validated those for EGFR and Histone H3.

To confirm successful gene editing (i.e., bioluminescence resulting from correct HiBiT insertion) and to determine the cell number to be used based on assay window (i.e., bioluminescent signal versus background) as well as to reduce reagent consumption in future Immuno-BRET experiments, we tested several cell numbers for HiBiT tag complementation by LgBiT. Based on our results, we selected 40,000 cells/well of EGFR-HiBiT-modified HeLa cells, 60,000 cells/well of STAT3-HiBiT-modified K562 cells, 40,000 cells/well of STAT3-HiBiT-modified LNCaP cells, 60,000 cells/well of MAPK8-HiBiT-modified K562 cells, 60,000 cells/well of c-MET-HiBiT-modified HeLa cells, and 1000 cells/well of HiBiT-H3F3-modified A-431 cells (Supplementary Fig. 1) for the next steps.

### Detection of phosphorylation on the receptor tyrosine kinases EGFR and c-MET

To demonstrate the utility of this approach, we initially focused on the detection of EGFR phosphorylation upon receptor activation. The epidermal growth factor receptor (i.e.: EGFR/ErbB1/HER1) family is the prototypical receptor tyrosine kinase which includes NEU/ERBB2/HER2, ERBB3/HER3, and ERBB4/HER4. Members of this family are capable of forming homo- and heterodimers and are critical to the normal embryogenesis of vertebrates^10^. These receptors are frequently upregulated in several cancers such as in metastatic colorectal cancer, head and neck cancer, pancreatic cancer, non-small-cell lung cancer, glioblastoma and breast cancer^11^.

**Figure 2:**
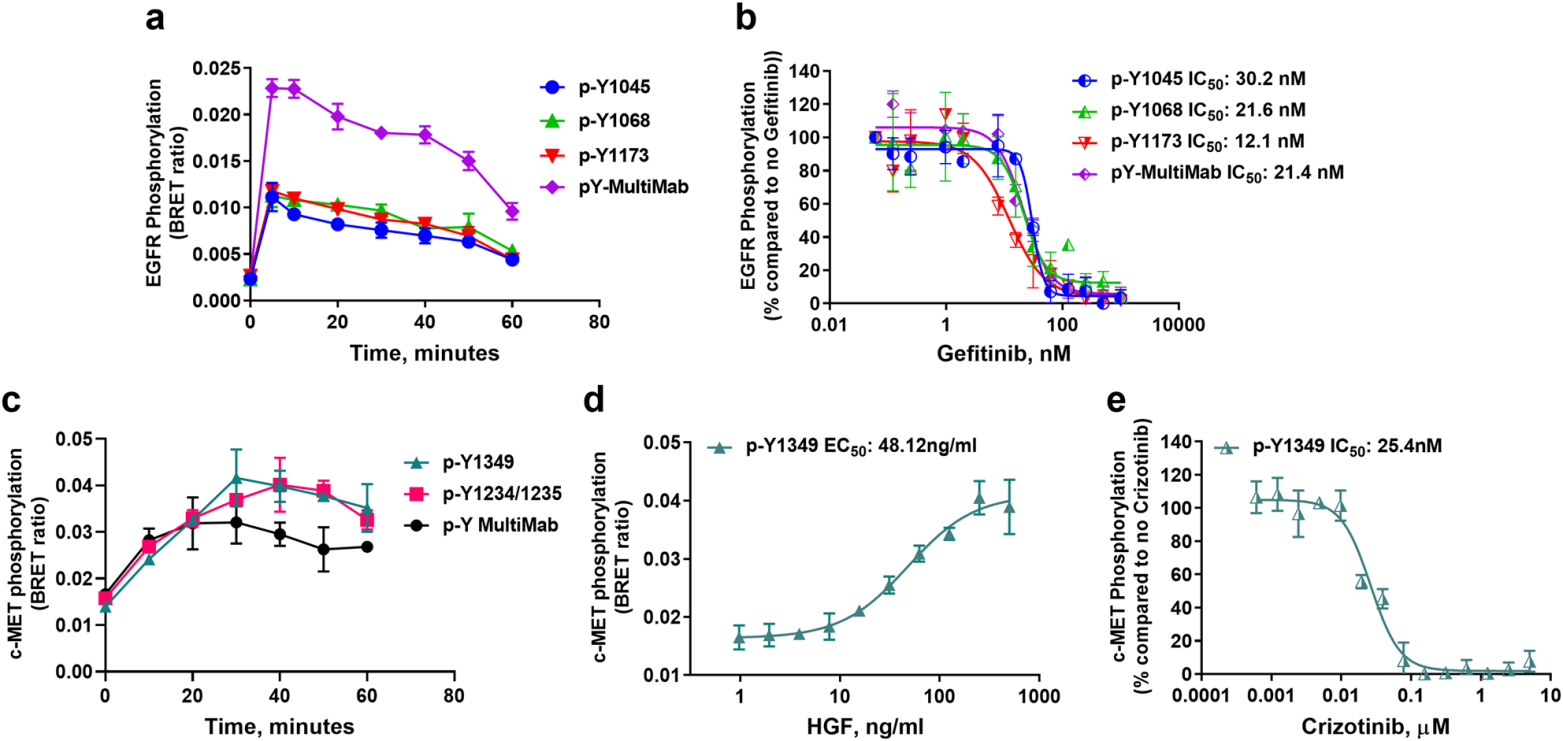
Receptor tyrosine kinase phosphorylation and inhibition detected with HiBiT-BRET approach using HiBiT/CRISPR-modified cells. (**a**) HeLa EGFR-HiBiT cells (40,000 cells/well) seeded overnight were treated the next day with 0.1 µg/mL EGF in different time points. (**b**) HeLa EGFR-HiBiT cells (60,000 cells/well) were treated with serially diluted gefitinib for 3 h at 37 °C. (**c**) HGF treatment time course of HeLa-c-MET-HiBiT cells. 0.1 µg/mL HGF was added to HeLa c-MET-HiBiT cells in different time points. (**d**) Serially diluted HGF was added to HeLa c-MET-HiBiT cells followed by incubation for 20 min at 37 °C. (**e**) Dose-dependent inhibition of c-MET-HiBiT phosphorylation with crizotinib. Serial dilutions of the inhibitor were incubated with modified cells for 3 h prior to addition of detection reagents. In all, after treatment, cells were lysed, and the HiBiT-BRET immunoassay was performed as described in “Materials and methods” section using the indicated antibodies. Results are representative of at least two independent experiments performed in duplicates.

EGFR is a single pass type I transmembrane receptor protein composed of 1210 amino acids subdivided into an extracellular domain (1–621 amino acids) responsible for interacting with soluble factors, a transmembrane domain (23 amino acids), and an intracellular domain (542 amino acids), which comprises a tyrosine kinase domain and a C-terminal tail containing various tyrosine residues that are phosphorylated upon receptor activation. The upregulation of EGFR activity is mediated through various mechanisms including mutations to its kinase domain (L858R and T790M mutations) and truncations such as the EGFRvIII truncations^11^. Several combinations of primary rabbit monoclonal anti-EGFR antibodies targeting different phospho-tyrosine residues with Alexa Fluor 594-labeled anti-rabbit secondary antibody were tested to detect EGF-dependent EGFR-HiBiT transphosphorylation through BRET in time course experiments. EGFR-HiBiT-edited HeLa cells were treated with 100 ng/mL EGF, and then lysed in the presence of the primary and secondary antibodies, LgBiT and furimazine. Our results indicated that all anti-phospho-tyrosine antibodies evaluated (anti-p-Y1045, anti-p-Y1068, anti-p-Y1173) were able to detect EGFR phosphorylation, with EGFR C-terminal tail phosphorylation peaking at 5 min upon EGF stimulation (Fig. 2A). Notably, the most pronounced results were observed with pan anti-phospho-tyrosine (P-Tyr-1000) MultiMab antibody mixture. It is noteworthy that we observed an 8–28% decrease in total bioluminescence values over the 60 min treatment with EGF (Supplementary Fig. 2A), suggesting that receptor levels have started to decrease with the EGF stimulation. This decrease agrees with the biology of EGFR and many other RTKs where activation upon ligand binding induces receptor internalization and endocytosis resulting in degradation or recycling^11^. Nevertheless, because the BRET signal is proportional to the amount of EGFR phosphorylation normalized to the tagged EGFR protein (BRET ratio), the BRET signal truly represents EGFR phosphorylation levels. Next, we evaluated the method’s sensitivity in measuring the effect of small molecule kinase inhibitors. The IC_50_ values obtained from CRISPR-modified EGFR-HiBiT HeLa cells treated with increasing concentrations of the EGFR kinase inhibitor gefitinib were similar across all anti-phospho-tyrosine antibodies tested, ranging from 12.07 to 30.19 nM (Fig. 2B, Table 1). These values were in agreement with reported IC_50_ value of 33 nM^12^. Given that EGFR has multiple tyrosines within its C-terminal tail that undergoes phosphorylation upon stimulation, the use of a monoclonal antibody mixture that targets multiple phospho-tyrosine residues likely improved the BRET signal compared to the EGFR site-specific anti-phospho-tyrosine antibodies. Also, total bioluminescence values remained stable across different gefitinib concentrations, suggesting that gefitinib did not affect cell viability and amount of HiBiT-tagged protein during the 1-h incubation period (Supplementary Fig. 2B).

**Table 1:** List of protein targets whose PTM level was detected with ImmunoBRET, and the corresponding experimental values obtained in this study.

Target	Size, kDa	PTM	Ligand	EC_50_	95% CI	Inhibitor	IC_50_, nM	95% CI
EGFR-HiBiT	170	p-EGFR (Y1045)	EGF	7.819* ng/mL	5.803–10.69*	Gefitinib	30.19	26.62–33.74
		p-EGFR (Y1068)		11.73* ng/mL	9.576–14.80*		21.57	15.21–31.12
		p-EGFR (Y1173)		23.32* ng/mL	17.27–36.96*		12.07	7.975–18.47
		P-Tyr-1000 MultiMab		14.61* ng/mL	12.90–16.74*		21.39	17.22–26.78
MET-HiBiT	84	p-MET (Y1349)	HGF	48.12 ng/mL	34.75–73.93	Crizotinib	25.4	21.51–32.41
STAT3-HiBiT_(K562)_	83–88	p-STAT3 (Y705)	IL-27	11.38 ng/mL	9.437–13.98	Ruxolitinib	6.919	4.928–9.338
STAT3-HiBiT_(LNCaP)_			IL-6	12.81 ng/mL	11.83–28.43		19.29	10.26–46.04
MAPK8-HiBiT	44–48	p-SAPK/JNK (T183/Y185)	TNF-α	1.617 ng/mL	0.6786–2.858	SP 600125	22,910	17,000–30,920
			Anisomycin	53.11 nM	4.884–65.50		N/A	N/A
HiBiT-H3F3A	15	Ac-H3K9	Trichostatin A	0.4583 µM	0.3436–0.6040	N/A	N/A	N/A
		Ac-H3K27		0.5363 µM	0.4052–0.7412	N/A	N/A	N/A

*Data not shown.

We also evaluated the Immuno-BRET method with the activation of a second receptor tyrosine kinase, c-MET as a potential viable method for inhibitor screening. The c-MET pathway regulates a diverse set of cellular processes, such as cell proliferation and angiogenesis. c-MET is a receptor tyrosine kinase, and acts on several downstream effectors (PLC-γ, c-Src) and pathways (PI3K/AKT, MAPKs, and Wnt), leading to different outcomes such as invasion, increased motility, proliferation, cell cycle progression and survival^13^. c-MET is a proto-oncogene; its aberrant activity is documented in several cancers, and its expression is associated with resistance to approved drugs, poor prognosis, and survival. It is considered a promising target, with drugs currently in preclinical and clinical phases^14–17^.

To detect c-MET phosphorylation as a result of HGF treatment, we evaluated commercially available phospho-tyrosine primary antibodies anti-p-MET (Y1349), anti-p-MET (Y1234/1235), and the pan anti-phospho-tyrosine (P-Tyr-1000) MultiMab in time-course experiments using c-MET-HiBiT-modified HeLa cells. All three antibodies tested detected c-MET-HiBiT phosphorylation in a similar manner; however, the MultiMab antibody mixture did not display a similar higher signal as was observed with this antibody for EGFR-HiBiT (Fig. 2C). Similar to total EGFR level decrease upon activation, we also observed a gradual decrease (10–20%) in total bioluminescence values after treatment with HGF longer than 20 min for c-MET, and therefore decided to limit stimulation to 20 min for the following experiments (Supplementary Fig. 2C). c-MET-HiBiT phosphorylation using different concentrations of HGF was also assessed using the anti-p-MET (Y1349) phospho-tyrosine primary antibody. Cells were incubated with serially diluted HGF for 20 min prior to the addition of detection reagents. Dose-dependent c-MET-HiBiT phosphorylation was observed with an estimated EC_50_ value of ~ 48.12 ng/mL HGF (Fig. 2D, Table 1). Finally, we evaluated whether this approach would have sufficient sensitivity toward detection of c-MET inhibitors by using crizotinib as a test compound^16^. Cells were treated with serially diluted crizotinib for 3 h, followed by stimulation using 100 ng/mL HGF for 20 min. The IC_50_ value observed was ~ 25.39 nM for crizotinib (Fig. 2E). A minor reduction of bioluminescence values was observed with higher concentrations of crizotinib, suggesting that the compound might have affected cell viability and amount of HiBiT-tagged protein in that concentration range (Supplementary Fig. 2E).

These results show that using CRISPR/Cas9-HiBiT-tagged cells, phosphorylation of receptor tyrosine kinases can be measured with the Immuno-BRET assay. The method is quantitative and sensitive enough to detect activation and inhibition of these kinases. Moreover, the findings show that a pan anti-Tyrosine antibody could be used as an alternative to specific site tyrosine phosphorylation when assessing RTK activation.

### Detection of phosphorylation on cytoplasmic targets using Immuno-BRET

We next evaluated the possibility of detecting intracellular target phosphorylation by using JAK-STAT pathway activation as a proof-of-concept. The mammalian JAK kinases and STAT transcription factors family members mediate the signal transduction cascade triggered by several cytokines. Activation of the JAK/STAT pathway begins at the plasma membrane through activation of surface receptors, resulting in gp130-/JAK-mediated tyrosine phosphorylation. The phosphorylation of specific tyrosine residues promotes STAT recruitment through their SH2 domains and subsequent phosphorylation by JAKs. This event results in STATs translocating to the nucleus, leading to the expression of genes involved in immune regulation and inflammation^18^. This pathway has been validated as an important therapeutic target for inflammatory disorders with drugs currently in the clinic.

**Figure 3:**
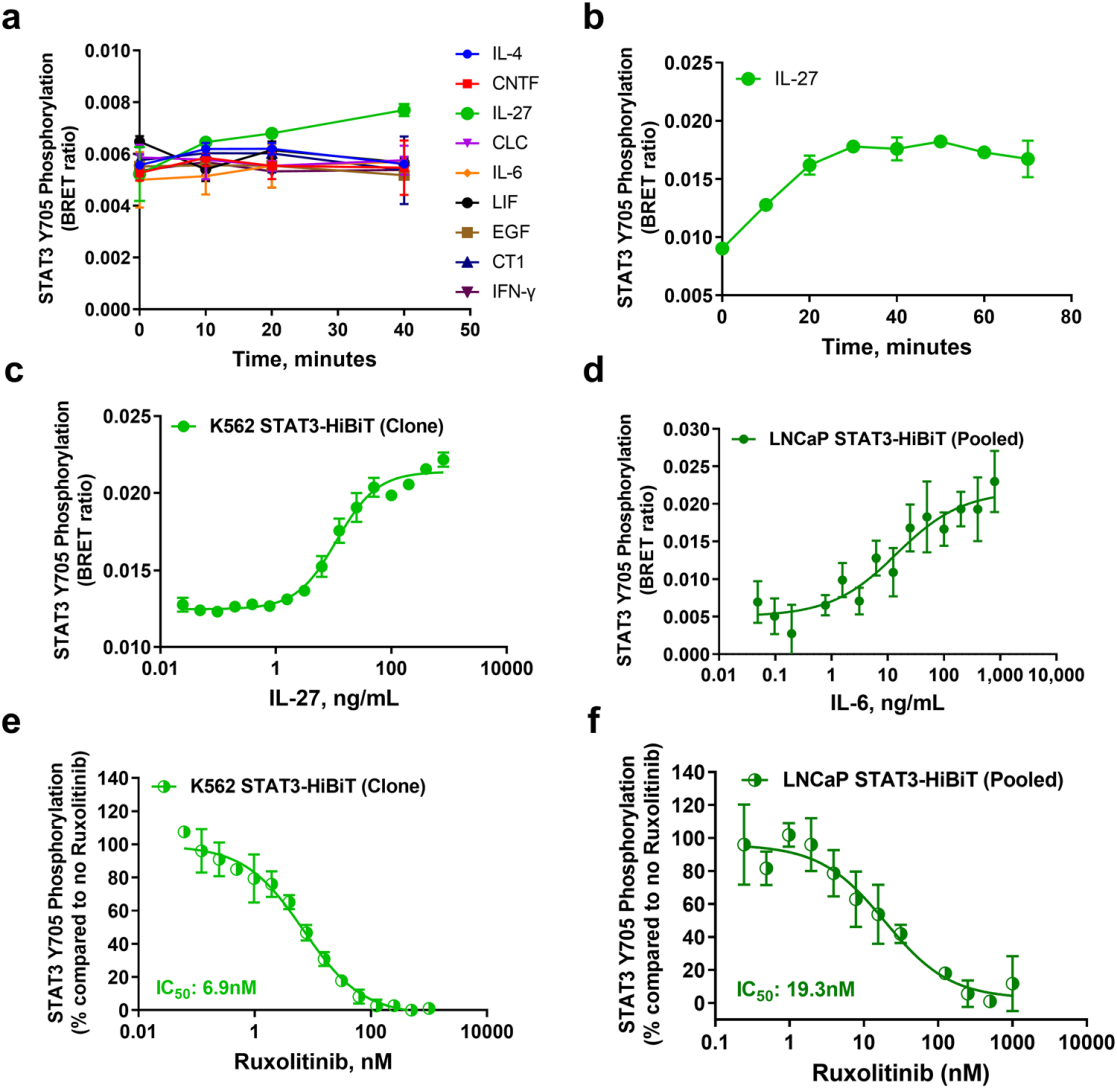
Stimulation and inhibition of STAT3 phosphorylation detected with HiBiT-BRET approach using HiBiT/CRISPR-modified cells. (**a**) Pooled K562 STAT3-HiBiT cells (60,000 cells/well) were treated with different cytokines at 0.1 µg/mL final concentration in different time points. (**b**) 60,000 pooled K562 STAT3-HiBiT cells seeded overnight were treated the next day with 0.1 µg/mL IL-27 every 10 min for 90 min. (**c**) K562 STAT3-HiBiT Cells (Clone) (60,000 cells/well) or (**d**) LNCaP STAT3-HiBiT pooled population cells (40,000 cells/well) were treated with serially diluted IL-27 or IL-6, respectively for 40 min at 37 °C. (**e**,**f**) Dose-dependent inhibition of STAT3 phosphorylation with ruxolitinib in K562 or LNCaP STAT3-HiBiT/CRISPR-modified cells. Results are representative of at least two independent experiments performed in duplicates.

Since it is well documented that K562 cells do not respond to IL-6 due to the lack of IL-6 receptor expression^19,20^, we first evaluated STAT3-HiBiT K562 cell response to a cytokine panel in the presence of anti-phospho-Stat3 (Tyr705) rabbit antibody or the pan anti-phospho-tyrosine (P-Tyr-1000) MultiMab, using a mixed population of edited cells. The anti-pY705 STAT3 antibody detected an increase in STAT-HiBiT phosphorylation in response to IL-27, whereas no changes were observed with the P-Tyr-1000 antibody (Fig. 3A and Supplementary Fig. 3A). In agreement with previous reports, IL-6 did not trigger STAT3 phosphorylation within 40 min.

We then determined STAT3 activation by IL-27 in time course experiments. 60,000 cells per well were treated with 100 ng/mL IL-27 and STAT3-HiBiT phosphorylation was detected using the anti-pY705 STAT3 antibody. STAT3-HiBiT phosphorylation was readily detected as soon as 10 min and reaches maximal phosphorylation at 40 min (Fig. 3B). Total bioluminescence values remained stable during treatment with IL-27 (Supplementary Fig. 3B). Next, to validate STAT3-HiBiT phosphorylation as a viable signaling node for monitoring the JAK/STAT pathway, cells were incubated with different concentrations of IL-27 for 40 min followed by lysis and addition of LgBiT, furimazine, and antibodies before bioluminescence and BRET were measured. We also isolated single clones expressing the STAT3-HiBiT and compared their bioluminescence generation to the mixed population of edited cells. Since the bioluminescence values generated by K562 STAT3-HiBiT isolated clones were higher compared with the mixed population of edited cells (Supplementary Fig. 3C), one of the clones was used for the IL-27 dose response experiment. A dose-dependent STAT3-HiBiT phosphorylation in K562 cells was observed, with apparent IL-27 EC_50_ value at ~ 11.38 ng/mL (Fig. 3C, Table 1). As a confirmation that STAT3 phosphorylation through IL-6 cytokine signaling can also be detected in another cell line (LNCaP) with Immuno-BRET, we also generated a mixed population of edited LNCaP-modified STAT3-HiBiT cells to demonstrate its phosphorylation upon IL-6 receptor activation. The EC_50_ value observed for IL-6 receptor-dependent STAT3-HiBiT activation in pooled LNCaP cells was ~ 12.81 ng/mL (Fig. 3D, Table 1).

To validate this approach as suitable for the detection of inhibitors for the JAK/STAT pathway, STAT3-HiBiT K562 clone cells and pooled STAT-3-HiBiT LNCaP cells were incubated with increasing concentrations of the JAK2 inhibitor ruxolitinib. As expected, ruxolitinib impaired STAT3 activation by JAK in a dose-dependent manner, with IC_50_ values at ~ 7 nM and ~ 20 nM for ruxolitinib in K562 and LNCaP cells respectively, in agreement with published literature (Figs. 3E,F and Table 1)^21^. It is noteworthy that both IL-27, IL-6 and ruxolitinib did not have any effect on cellular viability and amount of HiBiT-tagged protein as exemplified by the total bioluminescence values across the different data points (Supplementary Fig. 3D–G).

Another important signaling pathway evaluated with the Immuno-BRET approach was the stress-activated MAP kinase (MAPKs) pathway. MAPKs are serine/threonine kinases that play prominent roles in innate and adaptive immunity^22^. These kinases are activated through the phosphorylation of both threonine and tyrosine residues within the signature sequence –TXY–^23^. Overall, MAPK signal transduction is triggered either by receptor activation (i.e.: TLRs) or cellular stress, which leads to activation of an upstream MAPK kinase kinase (MAP3K), followed by phosphorylation of an intermediate MAPK kinase (MAP2K), and ultimately the activation of MAP kinases (MAPKs)^22^. Activated MAPKs phosphorylate multiple targets (i.e.: transcription factors) which in turn regulate gene transcription. There are four MAP kinase subfamilies: the extracellular signal-regulated kinases (ERK1/2), p38 (p38α/β/γ/δ), ERK5 and c-Jun NH_2_-terminal kinases (JNK-1/2/3)^22^.

**Figure 4:**
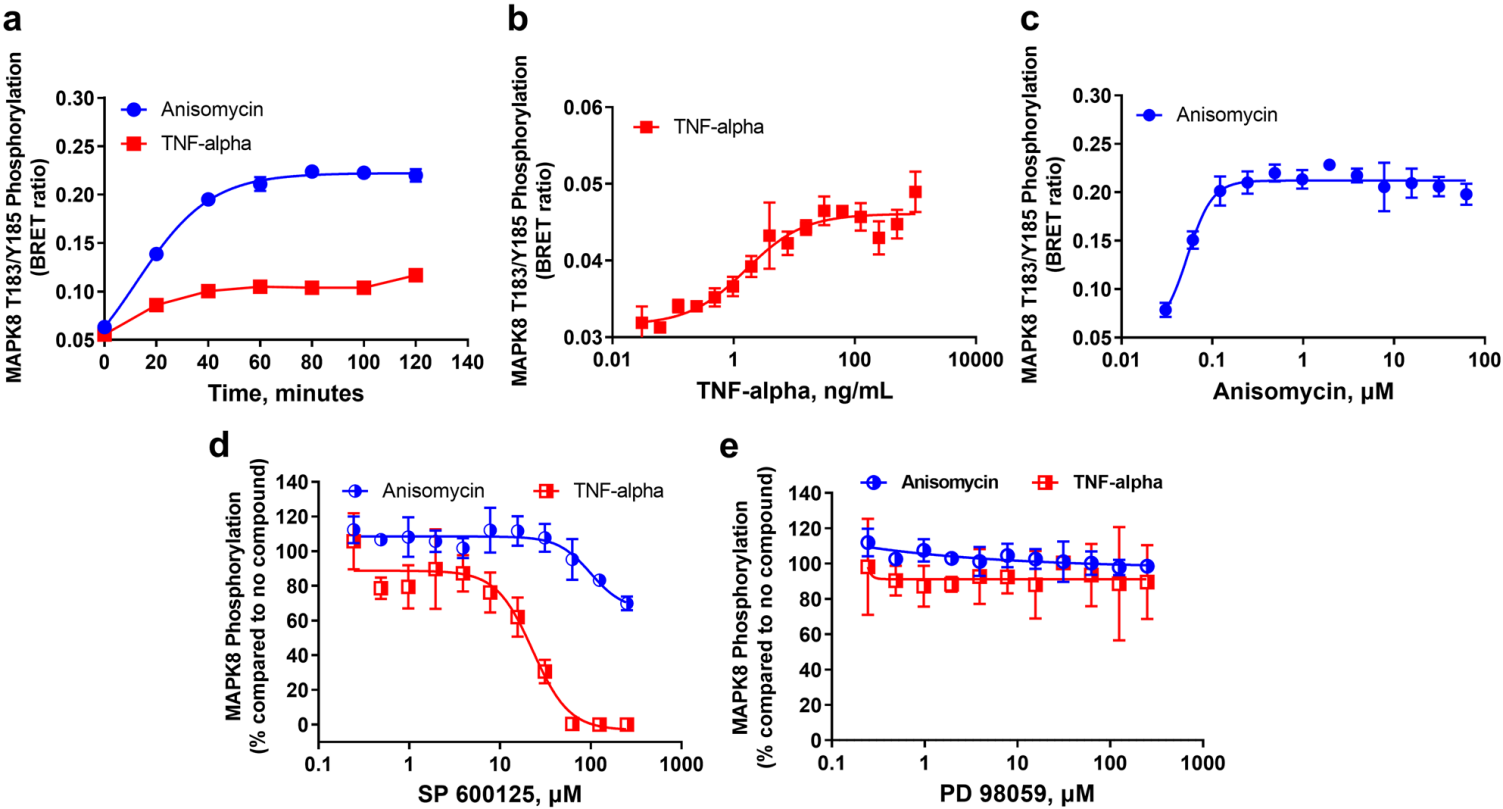
Stimulation and inhibition of MAPK8 phosphorylation using HiBiT/CRISPR-modified cells. (**a**) Time course phosphorylation of MAPK8 upon stimulation using 35 µM anisomycin or 0.1 µg/mL TNF-α every 10 min for 120 min (60,000 MAPK8-HiBiT-modified K562 cells/well). (**b**,**c**) Dose response of MAPK8 phosphorylation. Cells were treated with serially diluted TNF-α or anisomycin for 80 min at 37 °C. (**d**,**e**) Dose-dependent inhibition of MAPK8 phosphorylation. Serial dilutions of JNK inhibitor SP 600125 (D) or the MEK inhibitor PD 98059 were incubated with MAPK8-HiBiT CRISPR-modified cells for 3 h at 37 °C. Results are representative of at least two independent experiments performed in duplicates.

As a model for MAPK signaling activation, we started characterizing MAPK8 (i.e.: JNK-1) phosphorylation on Threonine 183 and Tyrosine 185 residues in K562 cells in response to treatment by different cytokines and small molecules (Supplementary Fig. 4A). As a control, MAPK8 phosphorylation was also stimulated by the known compound anisomycin and analyzed with Immuno-BRET. As expected, anisomycin triggered MAPK8 phosphorylation starting at 20 min treatment and leveling off at 60 min. We also observed MAPK8 phosphorylation upon treatment with TNF-α, indicating that MAPK8 activation is located downstream of TNFR stimulation. However, none of the other ligands tested stimulated this response (Supplementary Fig. 4A). We next assessed the time required to detect MAPK8 phosphorylation in a longer time course experiment by treating MAPK8-HiBiT-modified K562 cells with 100 ng/mL TNF-α or 35 µM anisomycin. We observed that MAPK8 reached maximal activation between 50–60 min as indicated by increased NanoBRET values using either treatment (Fig. 4A). Cell viability and amount of HiBiT-tagged protein appears to remain constant based on the total relative luminescence unit (RLU) values (Supplementary Fig. 4B,C).

We then evaluated the dose-dependent phosphorylation of MAPK8 upon TNF-α and anisomycin treatments. K562 cells expressing MAPK8-HiBiT were treated with different concentrations of TNF-α and anisomycin before performing the assay. As a result, the EC_50_ values observed were ~ 1.67 ng/mL and ~ 53 nM, respectively (Fig. 4B,C and Table 1). To validate MAPK8 Immuno-BRET assay as a valid method for detection of MAPK pathway inhibitors, we evaluated its sensitivity against different concentrations of the SAPK/JNK inhibitor SP 600125 and a control MEK inhibitor PD98059 followed by activation with TNF-α or anisomycin. The compound SP 600125 was capable of inhibiting MAPK8 phosphorylation in a dose-dependent fashion in the presence of TNF-α, with an IC_50_ of ~ 23 μM. However, MAPK8 phosphorylation was only partially inhibited in cells stimulated with anisomycin (Fig. 4D). As expected, no inhibition of MAPK8 phosphorylation was observed with the MEK inhibitor PD98059 (Fig. 4E). The total MAPK8 protein levels as assessed by NanoLuc complementation were similar across the different data points and treatments, indicating that the treatment did not have any effect on cell viability and amount of HiBiT-tagged protein (Supplementary Fig. 4B–D).

### Monitoring histone H3 acetylation using Immuno-BRET

**Figure 5:**
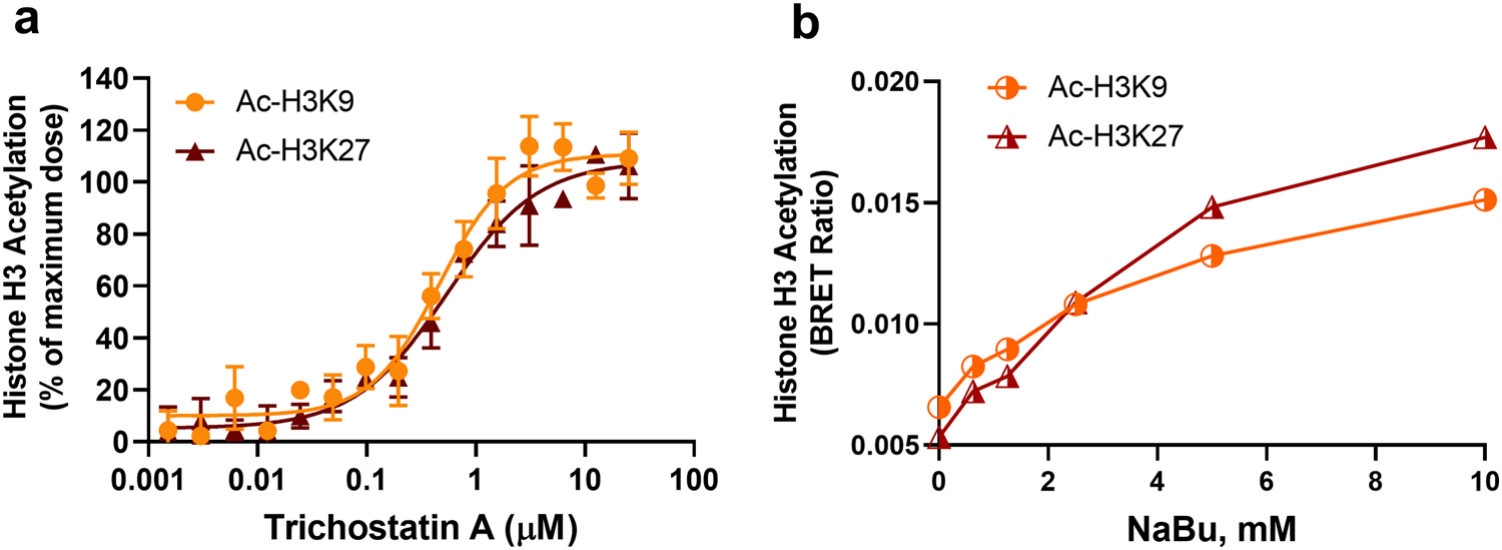
Detection of histone acetylation in A-431 H3F3A-HiBiT cells. (**a**) Serially diluted trichostatin A was added to CRISPR-modified cells (1000 cells/well) and incubated overnight at 37 °C. Histone acetylation was detected using anti-H3K9ac or anti-H3K27ac antibodies. (**b**) Histone H3 acetylation induced by sodium butyrate (NaBu). H3K9 and H3K27 acetylation was detected after incubation with sodium butyrate overnight at 37 °C. Results are representative of experiments performed in duplicates.

We demonstrated the feasibility of using the Immuno-BRET approach to measure phosphorylation levels of several targets and their regulation in selected disease-related pathways. To assess the applicability of this approach to other PTMs, we evaluated the feasibility of monitoring acetylation changes in HiBiT-histone H3 (i.e.: HiBiT tag was inserted at the N-terminus of histone H3 proteins) generated by CRISPR/Cas9 in A-431 cells. The equilibrium between acetylation and deacetylation of histones by histone acetyltransferases (i.e.: KATs) and histone deacetylases (i.e.: HDACs) is crucial for chromatin remodeling and gene expression in eukaryotes as well as metabolic pathways^24^. To monitor acetylation changes on histone H3 lysines 9 and 27, HiBiT-histone H3-modified A-431 cells were treated with different concentrations of trichostatin A (HDAC inhibitor), and the post-translational modifications were monitored using anti-histone H3AcK9 and anti-histone H3AcK27 antibodies. The results showed a dose-dependent accumulation of acetyl groups in both lysine residues due to HDAC inhibition, with EC_50_ values at around 0.45 µM and 0.53 µM for H3AcK9 and H3AcK27 acetylation levels, respectively (Fig. 5A). Treatment with another HDAC inhibitor, sodium butyrate (NaBu) also promoted acetylation of these residues (Fig. 5B), indicating that the HiBiT-BRET approach can also be applicable to acetylation detection.

## Summary

Overall, these results demonstrate the feasibility of monitoring changes in post-translational modifications of endogenous proteins through the combination of CRISPR/Cas9 technology for the introduction of bioluminescent reporter tags and the use of fluorescently labeled antibodies. The reporter tag (i.e.: HiBiT) allows the detection of dynamic changes associated with intracellular signaling, enabling the analysis of endogenous proteins while avoiding artifacts commonly observed in ectopic expression systems. Also, the application of CRISPR/Cas9 technology for detection of PTMs enables the evaluation of low abundance proteins with ease and speed not commonly seen in traditional immunoassays. We were able to monitor dynamic changes in phosphorylation upon activation and inhibition of pathways mediated by EGFR, c-MET, STAT3 and MAPK8, as well as observe changes in histone H3 acetylation in response to inhibition of histone deacetylases. The apparent IC_50_ values observed for the kinase inhibitors gefitinib, ruxolitinib, crizotinib were similar to earlier reports.

Traditional methods used to detect the level of PTMs in cell lysates include heterogenous ELISA and western blot-based assays, and homogenous HTRF- or AlphaLISA-based methods. Some of the weaknesses of those methods include low assay throughput, the requirement of multiple wash steps, lengthy protocols (4 h to overnight), and sometimes the need for special instruments. The Immuno-BRET assay is performed in a short time (2 h) and only requires a simple luminometer. As it is homogeneous, it does not require cell or cell media transfers or washing steps, and its “add and read” format enables detection of quick response signaling events. Also, the system is modular requiring only one specific primary and one universal secondary antibody pair for each target that could result in cost efficiency. Finally, monitoring PTM changes in endogenous targets avoids potential artifacts resulting from unregulated protein overexpression using expression vectors.

There are a few points that need to be considered when using HiBiT CRISPR-modified cells for post-translational modification studies. First, our results using K562 STAT3-HiBiT mixed population of edited cells and clones highlight the value of isolating edited cell clones to improve assay performance. Clone isolation maximizes signal intensity since it removes non-gene edited cells which could reduce the signal window. Therefore, it is advisable that cell sorting should be incorporated in the workflow in cases when low bioluminescence values are observed in cell pools.

Second, it is useful to test whether the use of an anti-phospho-tyrosine antibody for detection of a single or multiple p-Y sites within the target molecule will be advantageous compared with a site specific anti-phospho antibody. We observed opposing results when we tested the pan P-Tyr-1000 MultiMab in HeLa-EGFR-HiBiT and HeLa c-MET-HiBiT cells. Although both receptor tyrosine kinases exhibit an increase in tyrosine phosphorylation upon stimulation due to multiple Tyr phosphorylation sites, pan P-Tyr-1000 MultiMab showed better performance with EGFR. Conversely, monoclonal anti-phospho-target antibodies performed better with c-MET. Moreover, pan P-Tyr-1000 MultiMab was unable to detect the single STAT3 phosphorylation in K562 cells. While the use of the MultiMab anti-pY antibody can be beneficial for highly phosphorylated targets such as EGFR, it may not work to detect other tyrosine phosphorylated targets due to lower number of phospho-Tyrosines that are being detected or to the affinity of the MultiMab for the specific phospho-epitope of that target. Therefore, it is advisable to screen multiple antibodies in order to select which primary antibody is best suited for the target of interest. We have also incorporated this approach in other immunoassays that were devised for detection of endogenous targets^25^. Also, as previously demonstrated using the Frb/FKBP model system^26^, the energy transfer efficiency between the interacting proteins can be affected by geometric configuration. Likewise, the distance between the reconstituted NanoLuc—at either N- and C-terminus of the target protein—and its post-translational modification site(s) in our study likely affects energy transfer efficiency and BRET signal. Therefore, it is important to consider whether the proximity of the PTM sites to the reconstituted NanoLuc will enable BRET signal. Conversely, protein size alone may not restrict energy transfer as observed in our study. We successfully detected BRET signal from targets whose molecular weights ranged from 15 kDa (i.e.: H3F3A) to 170 kDa (i.e.: EGFR) (Table 1).

Third, it is imperative to understand the intracellular pathway of interest and if the target to be modified can be monitored in the cell line chosen for gene editing. Even though low expression levels could be a reason in some cases, pathway activation and consequently target phosphorylation could be dependent on the cellular context. This became clear with STAT3 activation in K562 cells, since this cell line lacks the IL-6 receptor and not surprisingly it was unresponsive to this cytokine.

Overall, combining Immuno-BRET approach with genome editing by CRISPR/Cas9 provides new means to monitor PTM changes by inserting reporters onto target endogenous genes while preserving native expression levels. Our results demonstrate its ability to efficiently monitor endogenous biological processes modulated by post-translational modifications using a small bioluminescent peptide tag and fluorescent antibodies in a homogeneous format, providing sensitive quantitation of the response dynamics to multiple stimuli.

### Supplementary Information

Supplementary Figures.

## Data Availability

The datasets used and/or analyzed during the current study are available from the corresponding author on reasonable request.
