# Supplementary material for: Monitoring phosphorylation and acetylation of CRISPR-mediated HiBiT-tagged endogenous proteins
Source: Sci Rep. 2024 Jan 25;14:2138. doi: 10.1038/s41598-024-51887-x (PMC10810970; doi:10.1038/s41598-024-51887-x)
Supplement: Supplementary file 1 — Supplementary Figures. [file 41598_2024_51887_MOESM1_ESM.pdf]

Monitoring phosphorylation and acetylation of CRISPR-mediated HiBiT-tagged endogenous proteins

Juliano Alves, Marie Schwinn, Thomas Machleidt, Said A. Goueli, James J. Cali, and Hicham Zegzouti.

Promega Corporation, R&D Department, 2800 Woods Hollow Road, Madison, WI 53711, USA

Correspondence and requests for materials should be addressed to:

Hicham Zegzouti, Ph.D. Phone: 608-443-3183. e-mail: hicham.zegzouti@promega.com

Juliano Alves, Ph.D. Phone: 608-443-3549. email: Juliano.alves@promega.com

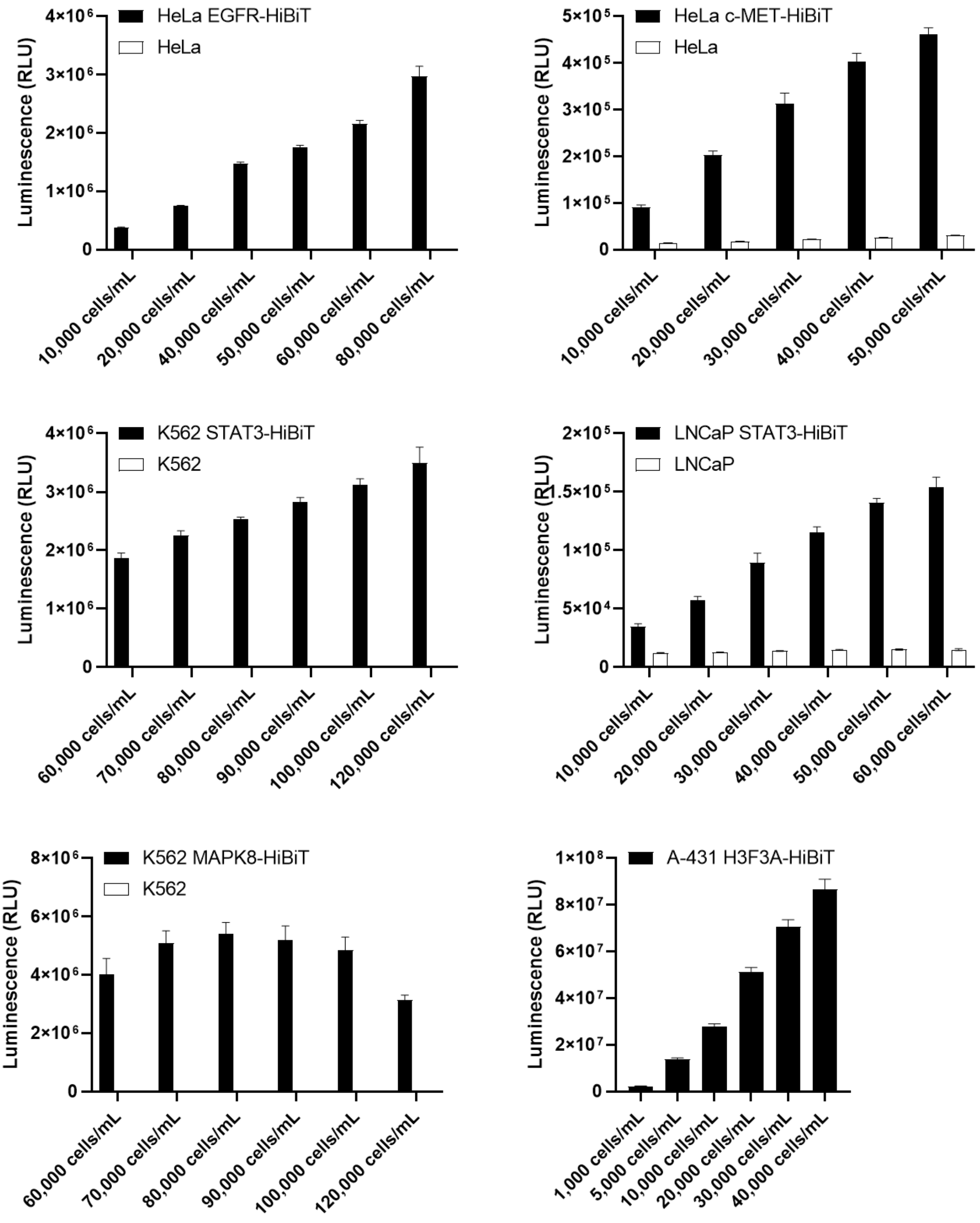

Supplementary Figure 1: Total bioluminescence values from dilutions of HiBiT-CRISPR-modified cell lines used in this study Results are representative of at least two independent experiments performed in duplicates.

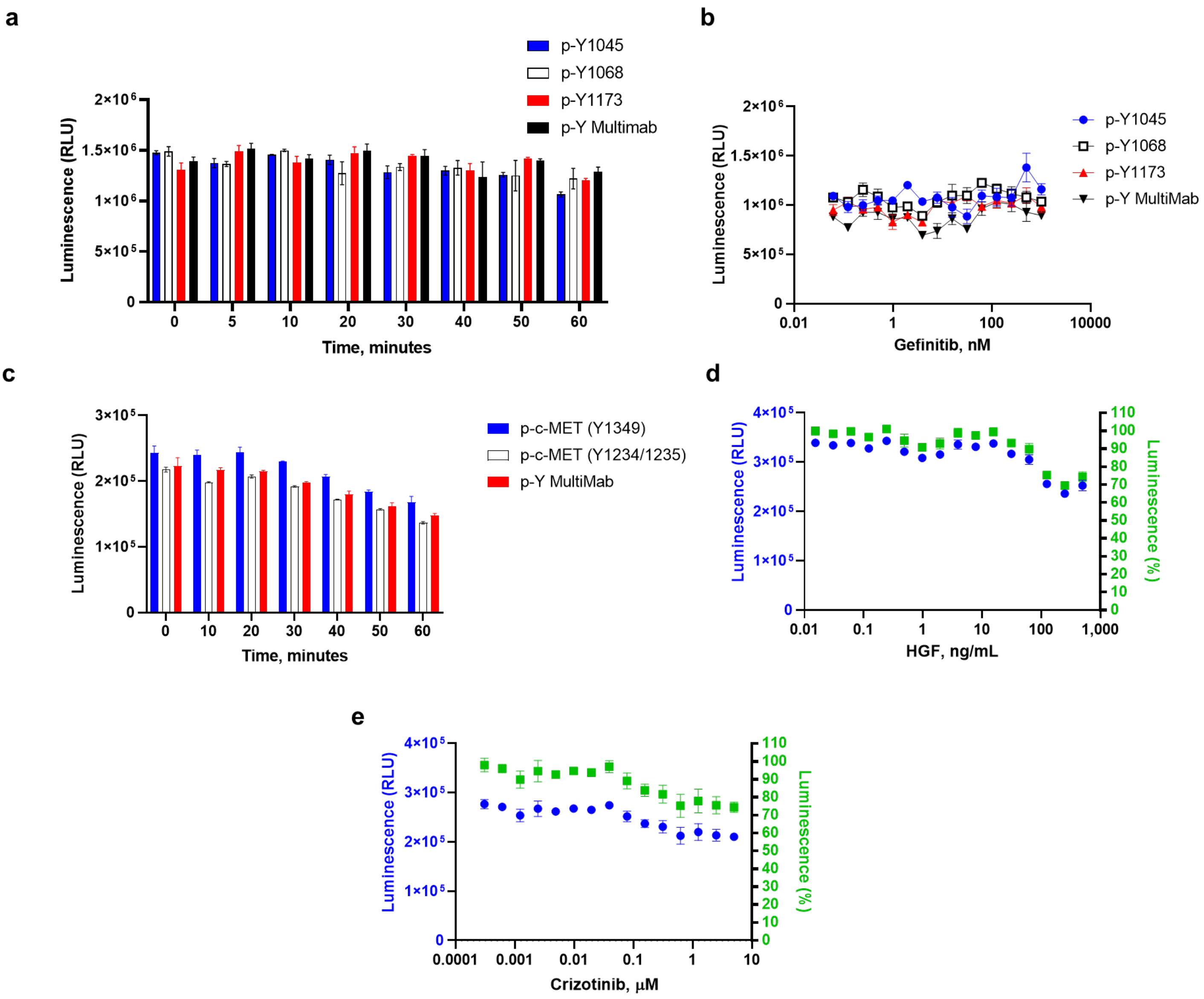

Supplementary Figure 2: Total bioluminescence values from HeLa EGFR-HiBiT cells depicted in figure 2. (**a**) EGF treatment and time course of HeLa EGFR-HiBiT (**b**) Gefitinib dose-response curve. (**c**) HGF treatment time course of HeLa-c-MET-HiBiT cells (**d**) HGF titration. (**e**) Crizotinib dose-response curve. Results are representative of at least two independent experiments performed in duplicates.

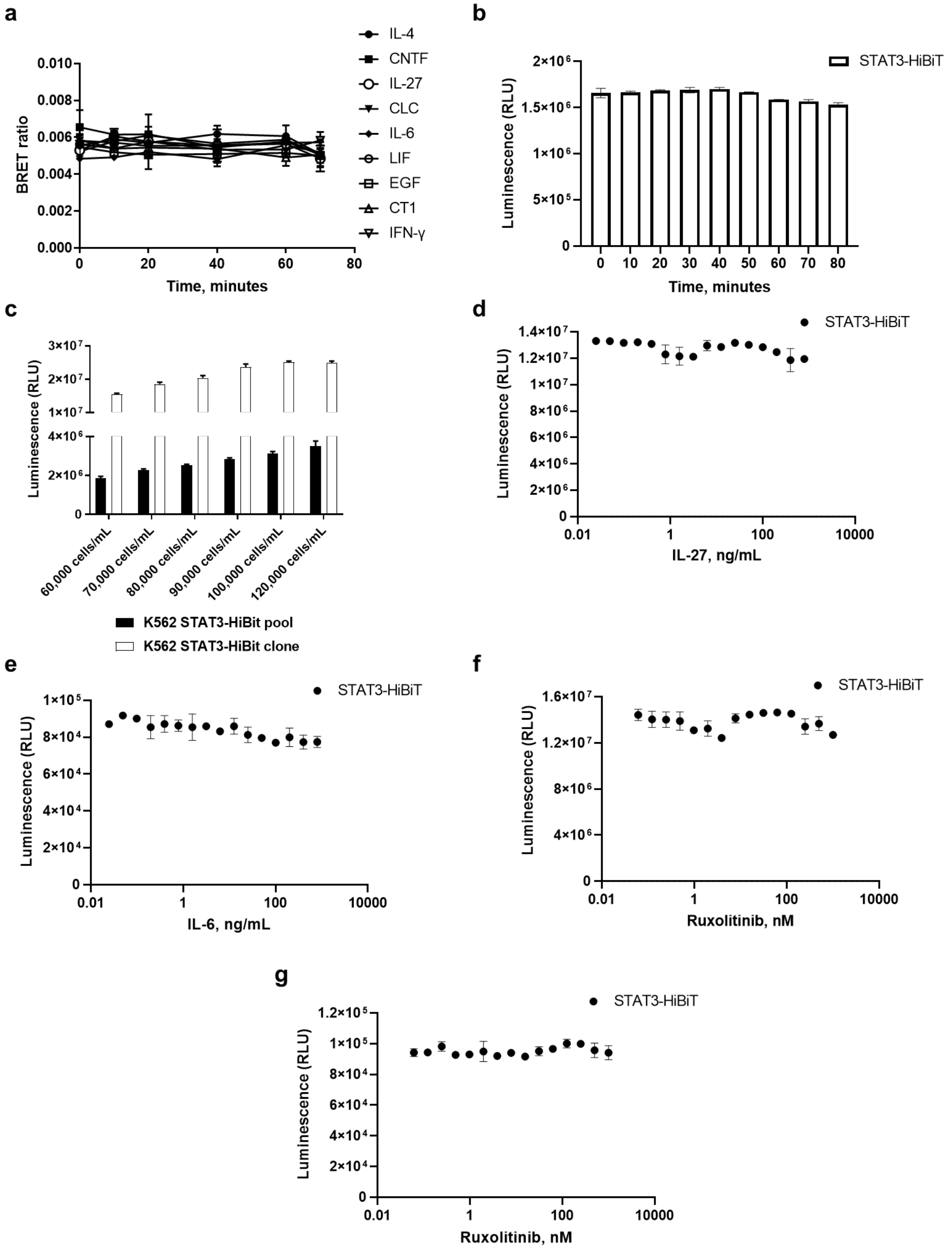

Supplementary Figure 3: Total bioluminescence values from K562 STAT3-HiBit and LNCaP STAT3-HiBiT cells depicted in figure 3. (**a**) Cytokine panel using P-Y-1000 MultiMab antibody mixture to detect STAT3 phosphorylation. (**b**) Total STAT3 generated bioluminescence during IL-27 treatment time course. (c) Bioluminescence generated by different number of K562 STAT3-HiBiT clones versus pooled cells. (**d, e**) Bioluminescence generated in the dose-response curves using IL-27 (K562 STAT3-HiBiT) (**d**) or IL-6 (LNCaP STAT3-HiBiT) (**e**) for 40 minutes at 37°C. (**f, g**) Dose-dependent inhibition of STAT3 phosphorylation in K562 (**f**) and LNCaP (**g**) HiBiT-CRISPR-modified cells using ruxolitinib. Results are representative of at least two independent experiments performed in duplicates.

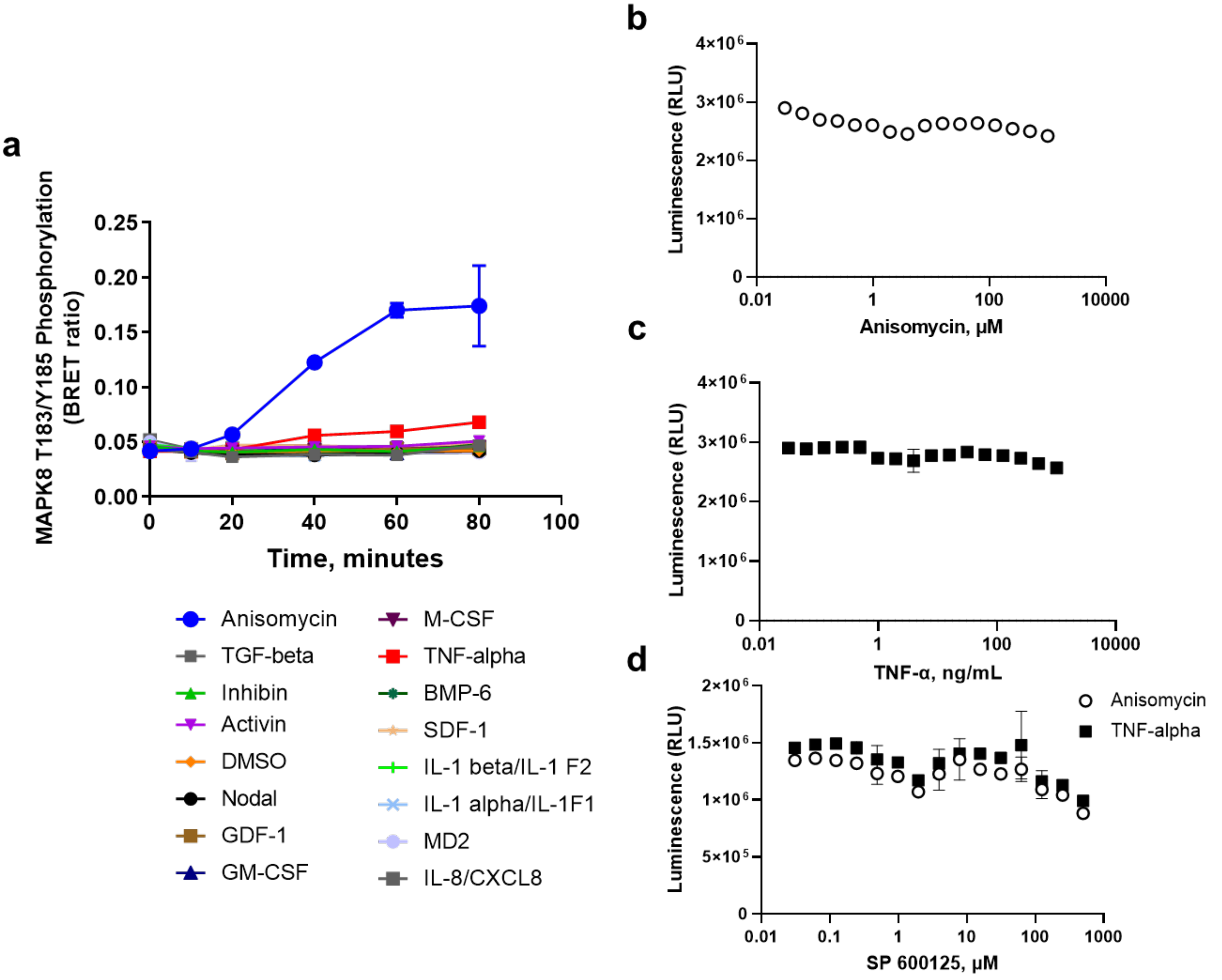

Supplementary Figure 4: Total luminescence values from K562 MAPK8-HiBit cells depicted in figures 4. (**a**) Cytokine panel and time course. (**b, c**) MAPK8 activation. Cells were treated with serially diluted TNF-α or anisomycin for 80 minutes at 37°C. (**d**) Dose-dependent inhibition of MAPK8 phosphorylation using serial dilutions of JNK inhibitor SP 600125. Results are representative of at least two independent experiments performed in duplicates.
